# P-1928. Socio-demographic Factors Associated With Aspergillus Azole Resistance

**DOI:** 10.1093/ofid/ofaf695.2097

**Published:** 2026-01-11

**Authors:** Cynthia Daniela Bazán Acevedo, Marco Villanueva Reza

**Affiliations:** Instituto Nacional de Enfermedades Respiratorias, Mexico City, Distrito Federal, Mexico; INSTITUTO NACIONAL ENFERMEDADES RESPIRATORIAS, MEXICO CITY, Distrito Federal, Mexico

## Abstract

**Background:**

Aspergillus is a globally distributed filamentous fungus that can cause a variety of clinical problems in humans. Azoles are the primary antifungal agents used to treat Aspergillus infections; however, in recent years, there has been a growing number of reports of Aspergillus species resistant to these compounds. Given the widespread use of azoles in agriculture, our main objective was to investigate the association between socio-demographic factors, such as rural living, and azole resistance in Aspergillus species.

**Figure:**
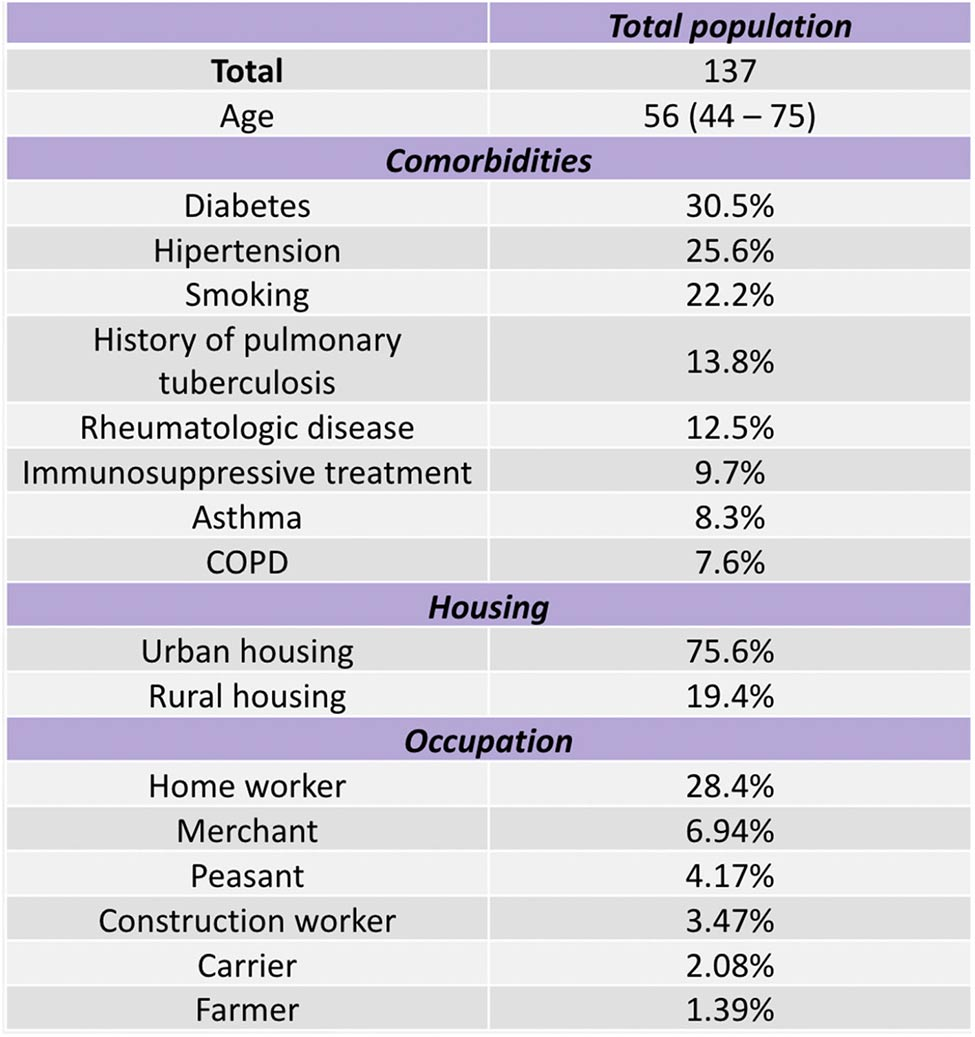
Clinical and Socio-demographic Characteristics of the General Population Table showing the mean age of the population, the main chronic comorbidities associated with Aspergillus disease, and the socio-demographic characteristics, including type of housing and predominant occupations.

**Figure:**
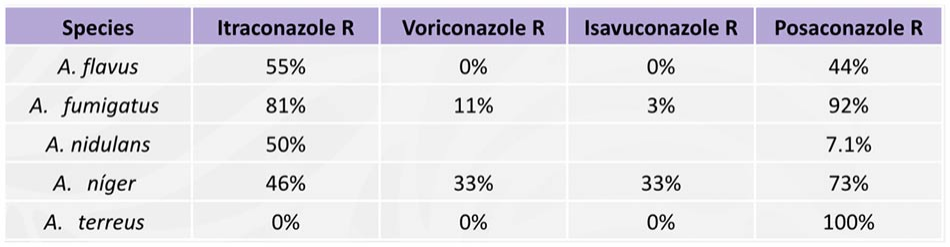
Prevalence of resistance in the isolated species R: Resistant. Table showing the isolated Aspergillus species and their resistance profiles to the most commonly used azoles for treatment.

**Methods:**

This was an observational, analytical, cross-sectional, retrospective case–control study. Cultures from respiratory samples with growth of Aspergillus spp. were evaluated. We included samples collected between January 1, 2020, and December 31, 2023. Resistance to various azole antifungals was assessed and categorized as resistant or susceptible. Sociodemographic data were then extracted from electronic clinical records, and samples were classified into four groups: rural living without occupational exposure, rural living with occupational exposure, urban living without occupational exposure, and urban living with occupational exposure. Odds ratios were calculated to determine potential sociodemographic associations with azole resistance in Aspergillus spp.

**Figure:**
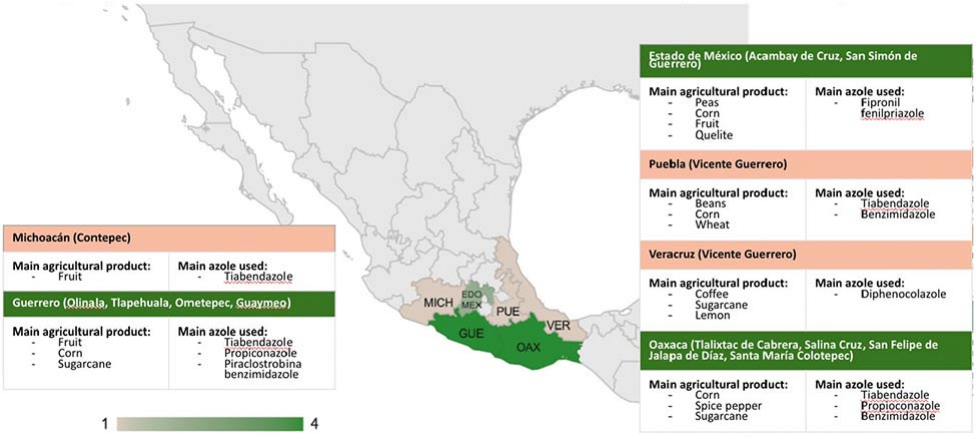
Geographic Distribution of Azole-Resistant Aspergillus Species in Mexico Figure showing the distribution of azole-resistant Aspergillus species in Mexico and its correlation with the main azole-based antifungals used in agriculture.

**Results:**

A total of 81 samples were included. A statistically significant association was found between rural living and voriconazole resistance, with the highest concentration of resistant isolates located in central Mexico. No significant associations were observed between other sociodemographic factors and resistance to the remaining azoles.

**Conclusion:**

Our study demonstrates that rural residency is significantly associated with the emergence of voriconazole-resistant Aspergillus. There results suggest that environmental use of azoles in agricultural settings may be driving resistance in clinical isolates. This finding is clinically important, as voriconazole is a first-line therapy for a range of Aspergillus-related syndromes, and environmentally acquired resistance may compromise its effectiveness

**Disclosures:**

All Authors: No reported disclosures

